# Integrating Osteology and Ancient DNA: Cranial Variation, Hemoglobin S, and Paternal Lineage in a Roman-Period Individual from Anatolia

**DOI:** 10.3390/life16060893

**Published:** 2026-05-26

**Authors:** Aylin Köseler, Ali Yalçın, İlker Kiraz, Gergana Lengerova, Martina Bozhkova, Steliyan Petrov, Ayla Sevim Erol

**Affiliations:** 1 Department of Biophysics, Faculty of Medicine, Pamukkale University, 20070 Denizli, Türkiye; 2 Milas Museum Director, Milas, 48200 Muğla, Türkiye; arthistorianaliyalcin@gmail.com; 3 Department of Neurosurgery, Faculty of Medicine, Pamukkale University, 20070 Denizli, Türkiye; ikiraz@pau.edu.tr; 4 Department of Medical Microbiology and Immunology “Prof. Dr. Elissay Yanev”, Medical University of Plovdiv, 4002 Plovdiv, Bulgaria; gergana.lengerova@mu-plovdiv.bg (G.L.); martina.bozhkova@mu-plovdiv.bg (M.B.); steliyan.petrov@mu-plovdiv.bg (S.P.); 5 Research Institute, Medical University of Plovdiv, 4002 Plovdiv, Bulgaria; 6 Center of Competence-Personalized Innovative Medicine, 4002 Plovdiv, Bulgaria; 7 Department of Anthropology, Faculty of Languages History and Geography, Ankara University, 06100 Ankara, Türkiye; aerol@ankara.edu.tr

**Keywords:** bioarchaeology, ancient DNA, third occipital condyle, HbS, Y-STR haplogroup, Anatolia

## Abstract

(1) Background: Integrated bioarchaeological approaches combining osteological and ancient DNA analyses provide powerful insights into health, disease, and population history in past societies. However, the relationship between rare skeletal variations, genetic disorders, and ancestry remains insufficiently explored within single individuals. This study aimed to investigate the combined osteological, paleopathological, and genetic characteristics of a Roman-period individual from southwestern Anatolia. (2) Methods: A multidisciplinary approach was applied to the skeletal remains of an adult male recovered from the Sekköy excavation site. Osteological analysis was conducted to assess cranial morphology, pathological lesions, and dental status. Ancient DNA was extracted from petrous bone under strict contamination control. The hemoglobin beta (HBB) gene was analyzed using Next Generation Sequencing and validated by Sanger sequencing. Y-chromosomal STR analysis was performed to determine paternal lineage. (3) Results: Osteological examination revealed a rare craniovertebral anomaly consistent with a third occipital condyle, along with porotic hyperostosis and extensive antemortem dental pathology, indicating prolonged physiological stress. Genetic analysis identified a heterozygous hemoglobin S mutation (HbAS; rs334), confirmed by both next-generation sequencing and Sanger sequencing, providing direct molecular evidence of hereditary hemoglobinopathy. Y-STR profiling assigned the individual to haplogroup R1b (predicted based on Y-STR data), indicating affiliation with Western Eurasian paternal lineages. (4) Conclusions: Despite the presence of comparable skeletal stress indicators, the integration of osteological and genetic data revealed a complex interaction between anatomical variation, chronic physiological stress, and inherited disease. The co-occurrence of a rare cranial anomaly, HbS mutation, and a defined paternal lineage represents a unique bioarchaeological case. These findings highlight the value of integrating skeletal and molecular approaches to reconstruct individual health profiles in archaeological contexts and demonstrate the methodological potential of interdisciplinary bioarcheological analysis.

## 1. Introduction

Human skeletal remains from archaeological contexts provide valuable insights into the biological characteristics, health status, and population history of past societies [1]. In recent decades, the integration of osteological analysis with ancient DNA (aDNA) research has significantly enhanced our ability to reconstruct individual life histories and investigate the genetic basis of disease and biological variation in antiquity [1,2]. This interdisciplinary framework enables a more comprehensive understanding of how genetic, environmental, and cultural factors have shaped human populations over time [2].

Cranial morphology represents one of the most informative aspects of human skeletal variation, reflecting a complex interplay of genetic inheritance, developmental processes, and environmental influences [3]. Among cranial structures, the occipital condyles play a crucial role in craniovertebral stability by articulating with the atlas to form the atlanto-occipital joint [4,5,6]. While their standard morphology is well documented, rare anatomical variants such as the third occipital condyle (condylus tertius) have been reported in both modern and archaeological populations [7,8]. Although often considered a non-pathological variation, this anomaly may have biomechanical and developmental implications and serves as an important marker of morphological diversity [7,8,9].

In addition to anatomical variation, skeletal indicators of physiological stress and disease are central to bioarchaeological interpretation. Porotic hyperostosis, characterized by porous lesions on the cranial vault, has traditionally been associated with iron-deficiency anemia but is now widely recognized as a multifactorial condition linked to nutritional deficiencies, chronic infections, parasitic burden, and hereditary hematological disorders [10,11,12]. Therefore, its presence should be evaluated within a broader biological and environmental context rather than attributed to a single cause.

Advances in ancient DNA methodologies have enabled the direct detection of genetic variants associated with disease in archaeological remains [13,14]. In particular, mutations affecting the hemoglobin beta (HBB) gene, such as hemoglobin S (HbS), are of major evolutionary and clinical importance [15,16]. HbS is associated with sickle cell disease and represents a well-established example of genetic adaptation to malaria, conferring a selective advantage in endemic environments [17]. The identification of HbS in ancient individuals provides critical evidence for the antiquity of hemoglobinopathies and their role in shaping human health and population dynamics in the Mediterranean and Near Eastern regions [17,18,19].

Furthermore, uniparental genetic markers, including Y-chromosomal short tandem repeats (Y-STRs), offer valuable insights into paternal lineage and population structure [20,21,22]. Haplogroup inference based on Y-STR data contributes to the reconstruction of genetic affinities, demographic processes, and patterns of continuity or mobility in past populations, particularly when interpreted within well-defined archaeological contexts [23].

Despite these advances, studies integrating rare cranial anatomical variation, skeletal indicators of physiological stress, and direct molecular evidence of hereditary disease within a single individual remain limited. In particular, the combined investigation of a third occipital condyle, porotic hyperostosis, and the HbS mutation in a Roman-period individual has not been previously documented in southwestern Anatolia.

The Sekköy excavation site, located in the Milas district of Muğla Province, represents a well-contextualized archaeological setting spanning the Hellenistic and Roman periods [24]. The human remains recovered from this site provide a unique opportunity to examine biological variation, disease, and genetic ancestry within a historically dynamic region characterized by long-term population interaction [24].

By combining skeletal observations with ancient DNA data, this research seeks to elucidate the interaction between anatomical variation, physiological stress, and genetic disease, while the present study aims to integrate osteological, paleopathological, and molecular genetic analyses to provide a comprehensive assessment of a single Roman-period individual from Sekköy. Rather than generating population-level conclusions, this study presents an integrative case analysis demonstrating the methodological value of combining skeletal and ancient DNA evidence in archaeological research.

**Figure 1:**
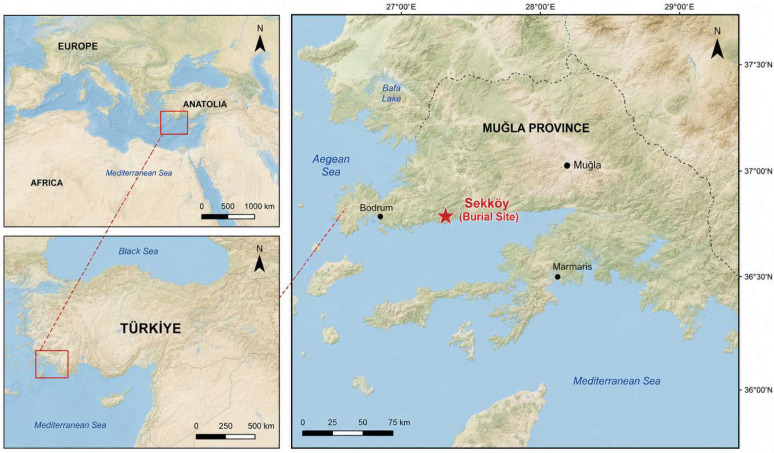
Geographical location of the Sekköy archaeological site in southwestern Anatolia (Muğla Province, Türkiye), showing its regional context within Anatolia and the eastern Mediterranean.

Figure 1 illustrates the geographical location of the Sekköy archaeological site within southwestern Anatolia and provides regional context for the analyzed burial.

## 2. Materials and Methods

### 2.1. Archaeological Context and Skeletal Material

The skeletal material analyzed in this study originates from the Sekköy excavation site, located in the Milas district of Muğla Province, southwestern Anatolia. Archaeological investigations conducted during the 2023–2024 field seasons revealed a burial area consisting of stone-built tombs primarily dated to the Hellenistic Period, with evidence of continued use into the Roman Period.

The tomb examined represents a multi-phase burial context and contained the disarticulated remains of five individuals deposited over successive burial events. The burial level was recorded at an elevation of 316.36 m above sea level, and individuals were oriented in northwest–southeast and southeast–northwest directions, consistent with local funerary practices. Repeated reuse of the tomb resulted in partial disturbance and disarticulation of skeletal elements.

Among the five individuals, one adult individual was selected for detailed analysis based on two primary criteria: (i) the presence of a clearly identifiable and rare craniovertebral anatomical variation consistent with a third occipital condyle, and (ii) sufficient skeletal preservation, including the petrous portion of the temporal bone suitable for ancient DNA analysis.

Skeletal elements attributed to this individual included cranial bones (particularly the occipital region and cranial vault), portions of the mandible and maxilla with associated dentition, and selected postcranial elements including pelvic fragments used for sex and age estimation. Element attribution was based on anatomical consistency, spatial proximity, preservation characteristics, and the absence of duplication of skeletal elements.

**Figure 2:**
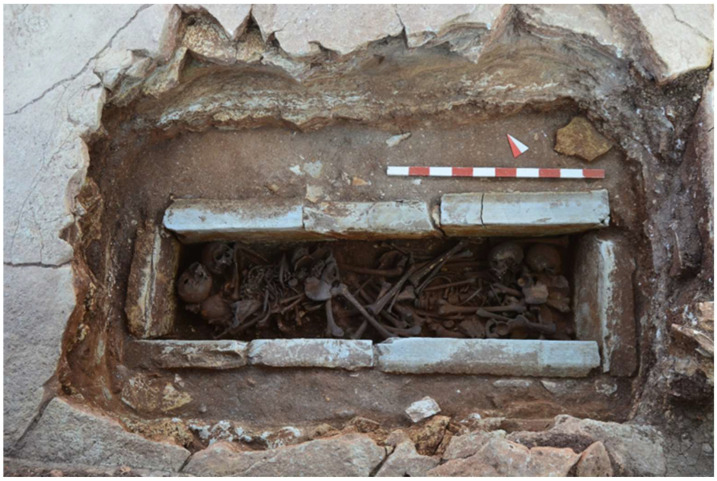
Spatial distribution and orientation of the five individuals identified within the Sekköy tomb. Skeletal elements are disarticulated due to repeated reuse of the burial context. Only anatomically consistent elements confidently attributed to the analyzed individual were included in the present study.

The remaining individuals recovered from the burial context were more fragmentary and did not exhibit comparable diagnostic anatomical features. In addition, preservation of dense skeletal elements suitable for ancient DNA analysis was insufficient in these individuals. Therefore, they were excluded from the integrated osteological and molecular analyses but are considered as part of the broader archaeological context. The spatial distribution and disarticulated nature of the skeletal remains are illustrated in Figure 2. The present study is designed as a methodological and integrative case study rather than a population-based analysis.

### 2.2. Osteological Analysis

Osteological analysis was conducted following internationally accepted standards in biological anthropology and paleopathology. Sex estimation was performed using morphological assessment of both pelvic and cranial features, following the methods described by Phenice [25] and Buikstra and Ubelaker [26]. Pelvic traits, including the subpubic angle, greater sciatic notch, and presence or absence of a preauricular sulcus, were prioritized due to their higher reliability. These observations were complemented by cranial characteristics such as the glabella, nuchal crest, supraorbital margins, and mental eminence.

Age-at-death estimation was primarily based on degenerative changes in the auricular surface of the ilium according to the method of Lovejoy et al. [27]. Dental wear was assessed as a secondary indicator using standard macroscopic scoring approaches; however, due to the disarticulated nature of the burial context, greater weight was given to pelvic age indicators when available.

All skeletal elements attributed to the analyzed individual were examined macroscopically under adequate lighting conditions. Only elements that could be confidently associated based on anatomical consistency and spatial proximity were included in the analysis. Particular attention was given to the craniovertebral junction, where the occipital condyles were carefully evaluated for morphological variation. A supernumerary osseous projection located adjacent to the occipital condyle was identified and interpreted as a third occipital condyle based on its anatomical position, morphology, and integration with the occipital bone.

Paleopathological assessment focused on identifying skeletal indicators of physiological stress and disease. Porotic lesions were recorded following established criteria in paleopathology. The presence, anatomical location, and macroscopic characteristics of porotic hyperostosis were documented on the cranial vault. The orbital roofs were examined for cribra orbitalia; however, no lesions consistent with this condition were observed in the preserved elements.

Dental analysis was conducted systematically following established osteological recording standards, including Buikstra and Ubelaker [26], adapted to the preservation status of the archaeological material. All preserved teeth and alveolar structures were recorded using the FDI World Dental Federation notation system. Antemortem tooth loss was identified by the presence of alveolar bone remodeling, while postmortem tooth loss was distinguished by the absence of such remodeling. Occlusal wear was assessed macroscopically using standard descriptive criteria. Additional dental pathologies, including caries, alveolar bone resorption, and possible periapical lesions, were recorded when observable. Antemortem tooth loss was identified by the presence of alveolar bone remodeling, while postmortem tooth loss was distinguished by the absence of such remodeling. Occlusal wear was assessed macroscopically and described in terms of severity. Additional dental pathologies, including caries, alveolar bone resorption, and possible periapical lesions, were recorded when observable. The degree of alveolar resorption was evaluated qualitatively to assess long-term oral health status.

The analysis also included evaluation of possible articular changes associated with the craniovertebral anomaly. The occipital condyle and adjacent articular surfaces were examined for evidence of degenerative changes, such as osteoarthrosis, which could indicate functional or biomechanical consequences of the anatomical variation. No definitive macroscopic evidence of advanced articular degeneration was observed in the preserved elements.

All observations were recorded descriptively, with emphasis on reproducibility and adherence to standard osteological protocols.

### 2.3. Ancient DNA Sampling and Contamination Control

All ancient DNA (aDNA) work was conducted in dedicated clean laboratory facilities designed for low-copy DNA analysis, physically separated from post-PCR laboratories. Strict contamination control measures were implemented throughout all pre-PCR procedures. Laboratory personnel wore full-body protective clothing, including disposable coveralls, face masks, hairnets, and double gloves. All work surfaces and equipment were routinely decontaminated using sodium hypochlorite (bleach) followed by ultraviolet (UV) irradiation. All procedures followed established guidelines for ancient DNA research, including strict contamination control and authentication criteria [28].

Sampling was performed on the petrous portion of the temporal bone, which is known to provide high endogenous DNA yield in archaeological specimens. Prior to sampling, the outer surface of the bone was mechanically removed using sterile rotary tools to eliminate potential modern contamination. The exposed inner portion was then UV-irradiated to further reduce surface contamination. Bone powder was obtained from the dense inner region of the petrous bone using sterile drills under controlled conditions.

DNA extraction was performed in small batches to minimize cross-contamination risk. Negative extraction controls (blanks) were included in each batch to monitor potential contamination during the extraction process. Similarly, no-template controls were incorporated in downstream amplification procedures.

Authentication of ancient DNA was assessed using multiple criteria. The extracted DNA exhibited characteristics typical of ancient DNA, including low concentration, short fragment length distribution, and molecular damage patterns such as elevated rates of cytosine-to-thymine (C → T) substitutions at the 5′ ends of DNA fragments. These features were evaluated during downstream sequencing analysis.

All procedures were performed in accordance with established guidelines for ancient DNA research, including recommendations to prevent contamination and ensure reproducibility. No modern human DNA was processed in the same laboratory space during the pre-PCR workflow.

These combined precautions and authentication criteria support the reliability of the obtained genetic data as originating from ancient biological material. Additionally, all procedures followed established ancient DNA guidelines [29,30].

### 2.4. DNA Extraction

DNA extraction was performed using protocols optimized for highly degraded and fragmented ancient DNA. Bone powder obtained from the petrous portion of the temporal bone was subjected to a decalcification step using 0.5 M ethylenediaminetetraacetic acid (EDTA) at room temperature with gentle agitation for an extended incubation period to ensure complete dissolution of the mineral matrix.

Following decalcification, DNA was extracted using a silica-based purification method, adapted from established ancient DNA protocols [31] and implemented using the QIAamp DNA Investigator Kit (QIAGEN, Hilden, Germany), with modifications to improve recovery of short DNA fragments. These modifications included prolonged lysis incubation and reduced elution volume to increase DNA concentration.

The extraction process relied on the binding of DNA molecules to silica membranes in the presence of chaotropic salts, followed by multiple wash steps to remove potential inhibitors and contaminants. DNA was eluted in a low-volume buffer suitable for downstream library preparation.

All extraction procedures were carried out in dedicated clean laboratory facilities under strict contamination control conditions. Negative extraction controls (extraction blanks) were included in parallel with each batch of samples to monitor potential contamination introduced during the extraction process.

The use of silica-based extraction methods specifically optimized for ancient DNA, combined with strict contamination controls, ensured efficient recovery of short, damaged DNA fragments characteristic of ancient biological material and provided a reliable template for downstream sequencing analyses.

### 2.5. NGS Library Preparation and Sequencing

Next Generation Sequencing (NGS) libraries were prepared using protocols optimized for ancient DNA, following established double-stranded library preparation methods [32]. Library preparation was performed using a partial uracil–DNA glycosylase (UDG) treatment (UDG-half protocol) to retain characteristic terminal damage patterns while reducing miscoding lesions within internal regions of DNA fragments.

Briefly, extracted DNA underwent end-repair to generate blunt-ended fragments, followed by adapter ligation using Illumina-compatible sequencing adapters. Unique dual indices were incorporated during library amplification to enable sample identification and to monitor potential cross-contamination.

Library amplification was carried out using a limited number of PCR cycles to minimize amplification bias and preserve library complexity. Amplified libraries were purified and quantified using fluorometric methods, and fragment size distribution was assessed to confirm the presence of short DNA fragments consistent with ancient DNA.

Sequencing was performed using Illumina paired-end technology. Raw sequencing reads were subjected to standard preprocessing steps, including adapter trimming, removal of low-quality reads, and filtering of short sequences.

The use of UDG-half treatment, combined with double-stranded library preparation, allowed reliable downstream analysis while preserving characteristic ancient DNA damage patterns necessary for authentication.

### 2.6. Bioinformatic Analysis and Variant Detection

Raw sequencing data were processed using a bioinformatic pipeline optimized for ancient DNA analysis. The analytical workflow and authentication criteria were implemented in accordance with current best-practice recommendations for ancient DNA studies [28]. Adapter sequences and low-quality bases were removed using AdapterRemoval v2, with reads shorter than 30 base pairs discarded to reduce noise and spurious alignments. Raw sequencing reads were processed using AdapterRemoval v2 for adapter trimming and quality filtering [33]. Filtered reads were aligned to the human reference genome (GRCh37/hg19) using the Burrows–Wheeler Aligner (BWA aln algorithm) [34]. Post-alignment processing, including sorting and duplicate removal, was performed using SAMtools (v1.17) [34] and Picard tools (v2.27.5) [35].

Ancient DNA authenticity was assessed using mapDamage software (v2.0 software) to evaluate characteristic nucleotide misincorporation patterns [36].

Filtered reads were aligned to the human reference genome (GRCh37/hg19) using the Burrows–Wheeler Aligner (BWA aln algorithm) with parameters adjusted for ancient DNA, including relaxed mismatch settings to accommodate post-mortem damage patterns.

Post-alignment processing included sorting, indexing, and removal of PCR duplicates using SAMtools and Picard tools to reduce amplification bias. Only uniquely mapped reads with high mapping quality were retained for downstream analysis.

Authentication of ancient DNA was assessed through the evaluation of characteristic damage patterns using mapDamage software, focusing on cytosine-to-thymine (C → T) substitutions at the 5′ ends and guanine-to-adenine (G → A) substitutions at the 3′ ends of DNA fragments.

Variant calling was performed on the HBB gene region using standard variant calling tools with stringent filtering criteria, including minimum read depth, base quality, and mapping quality thresholds. The HbS mutation was identified as a specific single nucleotide substitution within the HBB gene and was supported by multiple independent reads. Primer sequences are provided in Supplementary Table S1.

All analyses were conducted using widely accepted tools and parameters for ancient DNA research, ensuring reproducibility and reliability of the detected variants. The nucleotide sequence generated in this study has been submitted to GenBank under the accession number PZ306518.

### 2.7. Sanger Sequencing for HbS Validation

To validate the HbS mutation identified through Next Generation Sequencing, targeted Sanger sequencing was performed on the HBB gene region encompassing the known mutation site (codon 6, rs334).

PCR amplification was carried out using primers specifically designed to target a short fragment of the HBB gene suitable for degraded ancient DNA. Amplicon length was kept below 150 base pairs to ensure efficient amplification of fragmented DNA. Primer sequences were designed based on the human reference genome (GRCh37) and are provided in Supplementary Table S1.

PCR reactions were performed in a dedicated clean environment using optimized conditions for ancient DNA, including increased cycle numbers and the use of high-fidelity DNA polymerase. Negative PCR controls were included in all amplification reactions to monitor contamination.

PCR products were visualized on agarose gels to confirm successful amplification and appropriate fragment size. Successful amplicons were purified prior to sequencing.

Sanger sequencing was performed using standard dye-terminator chemistry on an automated capillary electrophoresis platform. Sequence chromatograms were manually inspected to verify base calls at the mutation site.

The presence of the HbS mutation was confirmed by identifying the characteristic single nucleotide substitution (A > T) at codon 6 of the HBB gene. Concordance between NGS and Sanger sequencing results was used to validate the reliability of the detected variant.

### 2.8. Y-Chromosomal STR Analysis

Y-chromosomal short tandem repeat (Y-STR) analysis was performed to investigate the paternal lineage of the analyzed individual. Amplification of Y-STR loci was carried out using the AmpFlSTR^®^ Yfiler™ PCR Amplification Kit (Applied Biosystems, Foster City, CA, USA), which targets a standardized panel of Y-STR markers widely used in both forensic and population genetic studies.

PCR amplification was conducted following the manufacturer’s protocol, with minor modifications to accommodate the degraded nature of ancient DNA. These modifications included a limited increase in PCR cycle number to enhance amplification success while minimizing stochastic effects. Reaction conditions were optimized to maintain specificity and reproducibility.

All amplification steps were performed in dedicated clean laboratory environments under strict contamination control measures. Negative PCR controls were included in each amplification batch to monitor potential contamination. No amplification was observed in negative controls.

To further assess potential contamination, the obtained Y-STR profiles were compared against available laboratory personnel profiles where applicable, ensuring that no matches were observed. This step provided an additional level of authentication for the generated genetic data.

Amplified PCR products were separated using capillary electrophoresis on an automated genetic analyzer. Allele sizes were determined by comparison with an internal size standard, and allele calling was performed using appropriate genotyping software following standard analytical thresholds.

Given the challenges associated with ancient DNA, particular care was taken to interpret allelic profiles conservatively, considering the possibility of allelic dropout and stochastic effects. Only reproducible and clearly defined alleles were included in the final haplotype.

### 2.9. Haplogroup Inference

The Y-STR haplotype obtained from the analyzed individual was used to infer the most likely Y-chromosomal haplogroup through comparison with established reference databases and prediction tools that correlate Y-STR profiles with known Y-SNP-defined haplogroups.

Haplogroup assignment was performed using publicly available Y-STR haplogroup prediction algorithms and databases. The inferred haplogroup was determined based on the closest match between the observed STR profile and reference haplotypes.

It is important to note that haplogroup inference based solely on Y-STR data has inherent limitations and may not provide the same level of resolution and accuracy as direct Y-SNP analysis [37]. Therefore, the assigned haplogroup should be considered a probabilistic estimation rather than a definitive classification.

Despite these limitations, Y-STR-based haplogroup prediction has been shown to provide reliable approximations of paternal lineage when interpreted cautiously and within an appropriate archaeological and population genetic context.

Accordingly, the inferred haplogroup in this study is interpreted conservatively and used to provide general insight into paternal lineage affiliation rather than precise phylogenetic placement.

## 3. Results

### 3.1. Osteological Findings

The analyzed skeletal remains were attributed to an adult male individual based on morphological assessment of pelvic and cranial features. Age-at-death estimation, primarily derived from auricular surface morphology and supported by dental wear, indicates an age range of approximately 30–34 years.

Macroscopic examination of the cranial vault revealed the presence of porotic hyperostosis localized predominantly on the parietal bones. The lesions exhibited a porous, trabecular appearance consistent with remodeled diploë expansion. No evidence of cribra orbitalia was observed in the preserved orbital roofs.

**Figure 3:**
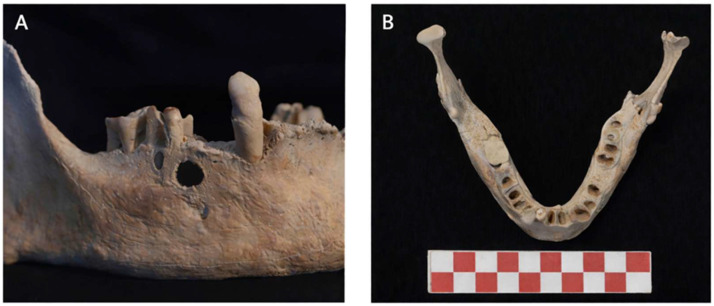
Dental and alveolar pathology in the analyzed individual. (**A**) Lateral view of the mandible showing extensive antemortem tooth loss (AMTL) accompanied by marked alveolar bone resorption. (**B**) Superior view of the mandible illustrating multiple empty alveoli and advanced occlusal wear on the remaining teeth.

Differences observed between mandibular views (Figure 3A,B) are attributable to postmortem damage, fragmentation, and displacement within the burial context rather than representing different individuals.

**Figure 4:**
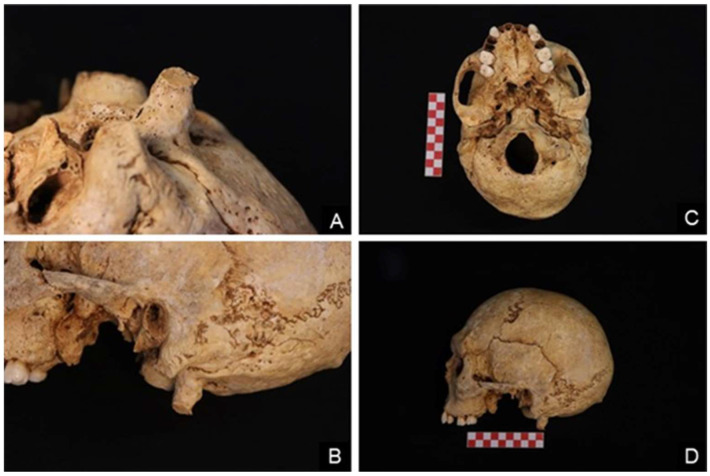
Osteological features observed in the analyzed individual. (**A**,**B**) Supernumerary osseous projection consistent with a third occipital condyle located on the left lateral aspect of the occipital bone, shown from different perspectives to illustrate its morphology and anatomical continuity. (**C**) Mandibular and maxillary elements demonstrating antemortem tooth loss and alveolar bone resorption. (**D**) Porotic hyperostosis on the cranial vault, characterized by porous lesions and diploë expansion. All features are presented at the macroscopic level. The observed patterns reflect a combination of anatomical variation, chronic physiological stress, and dental pathology.

Examination of the craniovertebral junction identified a supernumerary osseous projection on the left lateral aspect of the occipital bone, consistent with a third occipital condyle (Figure 4A,B). The structure showed clear anatomical continuity with the occipital bone. No macroscopic evidence of advanced degenerative joint disease was observed. Dental findings, including antemortem tooth loss and alveolar resorption, are illustrated in Figure 4C. Porotic hyperostosis observed on the cranial vault is shown in Figure 4D.

Dental analysis revealed severe and widespread pathology. A total of 18 alveolar positions could be assessed. Of these, 11 teeth were lost antemortem, as indicated by clear alveolar bone remodeling, while 4 teeth were preserved in situ. The remaining alveoli showed postmortem tooth loss.

The preserved teeth exhibited advanced occlusal wear, consistent with prolonged functional use. Marked alveolar bone resorption was observed in both the maxilla and mandible, indicating chronic periodontal disease. In addition, observable dental conditions included probable carious lesions and irregular alveolar morphology suggestive of long-term oral pathology. No definitive periapical abscesses could be confirmed macroscopically.

Overall, the osteological findings indicate a combination of chronic physiological stress, significant dental deterioration, and a rare craniovertebral anatomical variation in the analyzed individual. In addition to the findings described above, no further rare cranial or postcranial anatomical variants were observed in the analyzed individual.

Macroscopic examination of the preserved skeletal elements did not reveal clear evidence of chronic infectious disease, such as periosteal reactions, lytic lesions, or cloaca formation. This suggests that, despite the presence of physiological stress indicators, there is no direct osteological evidence for chronic systemic infection.

The four remaining individuals recovered from the same burial context were assessed for comparable craniovertebral anatomical variation, and no additional examples of a third occipital condyle were identified (0/4). Accordingly, this anatomical finding was limited to the single analyzed individual.

Observed differences between panels are attributable to postmortem damage, fragmentation, and displacement within the burial context rather than representing different individuals. Overall, the dental and alveolar changes are consistent with chronic physiological stress and long-term oral pathology.

### 3.2. Next Generation Sequencing Results

Next Generation Sequencing (NGS) analysis yielded DNA fragments suitable for molecular investigation of the archaeological specimen. Sequencing generated a total of 142,061,360 raw reads, of which 135,400,927 (98.42%) were successfully mapped to the human reference genome, and 130,272,327 (96.21%) remained uniquely mapped after quality filtering and duplicate removal. The average sequencing coverage across the analyzed target regions was 136.6×.

Sequencing quality assessment demonstrated high-quality data, with Q20 and Q30 values of 97.93% and 93.92%, respectively, and an effective post-processing data yield of 82.44%. Ancient DNA authentication analysis demonstrated characteristic terminal nucleotide misincorporation patterns consistent with authentic ancient DNA, as assessed by mapDamage analysis. Additional authentication and sequencing quality metrics are provided in Supplementary Table S3.

Targeted analysis of the HBB gene region identified the rs334 variant corresponding to the hemoglobin S (HbS) mutation. The rs334 locus was covered by 38 independent sequencing reads, including 18 reads supporting the mutant HbS allele and 20 reads supporting the wild-type allele, consistent with a heterozygous HbAS genotype. This finding was independently confirmed by Sanger sequencing.

The nucleotide sequence generated in this study has been deposited in the GenBank database under accession number PZ306518.

### 3.3. Sanger Sequencing Validation

Targeted Sanger sequencing successfully confirmed that the detected HbS mutation was present in heterozygous form (HbAS, sickle cell trait), indicating one mutant and one wild-type allele rather than homozygous HbSS sickle cell disease. The mutation was confirmed to be present in a heterozygous state (HbAS), as indicated by the presence of dual peaks at the mutation site in the Sanger sequencing chromatogram, corresponding to both allelic variants.

Amplification of the HBB gene region encompassing codon 6 yielded clear and reproducible PCR products. Sequencing chromatograms demonstrated a distinct nucleotide substitution corresponding to the HbS mutation (rs334), confirming the presence of the A > T transversion. The observed sequence variation was consistent across independent sequencing reactions, and chromatogram quality allowed unambiguous base calling at the mutation site. The agreement between NGS and Sanger sequencing results provides strong validation of the detected HbS mutation and supports the reliability of the molecular findings.

### 3.4. Y-STR Analysis and Haplogroup Assignment

Y-chromosomal short tandem repeat (Y-STR) analysis was performed using the AmpFlSTR^®^ Yfiler™ PCR Amplification Kit (Applied Biosystems, Foster City, CA, USA), targeting a standardized panel of Y-STR loci.

PCR amplification was carried out following the manufacturer’s protocol, with modifications optimized for degraded ancient DNA. Specifically, the number of PCR cycles was increased from the standard 28 cycles to 32 cycles to improve amplification success while minimizing stochastic effects such as allelic dropout and preferential amplification. Reaction volumes and reagent concentrations were maintained according to the manufacturer’s recommendations.

Thermal cycling conditions consisted of an initial denaturation step at 95 °C for 11 min, followed by 32 cycles of denaturation at 94 °C for 1 min, annealing at 61 °C for 1 min, and extension at 72 °C for 1 min, with a final extension step at 60 °C for 80 min.

All amplification procedures were performed in dedicated clean laboratory environments under strict contamination control measures. Negative PCR controls were included in each amplification batch, and no amplification was observed in these controls.

To further assess potential contamination, the obtained Y-STR profiles were compared against available laboratory personnel reference profiles, and no matches were identified. This provides additional support for the authenticity of the generated genetic data.

Given the degraded nature of ancient DNA, allelic profiles were interpreted conservatively. Only reproducible alleles observed across independent amplifications were retained for haplotype construction. The complete Y-STR haplotype is provided in Supplementary Table S2.

## 4. Discussion

The present study provides an integrated bioarchaeological and paleogenetic assessment of a single Roman-period individual from the Sekköy site in southwestern Anatolia [24]. As an individual case study, the findings should be interpreted cautiously and not as representative of broader population-level biological patterns.

A key finding of this study is the coexistence of porotic hyperostosis and a genetically confirmed hemoglobin S (HbS) mutation. This combination supports a cautious multifactorial interpretation of cranial porosity, without implying a direct causal relationship between the heterozygous HbS mutation and the observed skeletal lesions. While porotic hyperostosis has traditionally been linked to iron-deficiency anemia [11], increasing evidence suggests that it reflects complex interactions between nutrition, infection, and genetic factors [10,12,19]. The identification of the HbS mutation provides relevant biological context for interpreting the analyzed individual; however, because the mutation was identified in heterozygous form (HbAS), corresponding to sickle cell trait rather than sickle cell disease, it should not be considered a direct explanation for the observed skeletal lesions [17,19].

The identification of a third occipital condyle represents a rare but informative example of craniovertebral anatomical variation. Although generally considered non-pathological, such variations may have biomechanical implications. The third occipital condyle (condylus tertius) represents a rare anatomical variation in the craniovertebral junction, generally considered to arise from developmental anomalies during ossification of the occipital region. Previous anatomical studies have reported its prevalence to be low in both modern and archaeological populations, typically below 1%, highlighting its rarity. Although often classified as a non-pathological variant, its presence may influence the biomechanics of the atlanto-occipital joint, potentially altering articulation patterns or load distribution. In archaeological contexts, such variations are particularly informative as markers of morphological diversity and developmental variation rather than disease. The identification of this feature in the present individual therefore contributes to the limited body of evidence documenting craniovertebral anatomical variability in past populations. Clinical and anatomical studies have associated occipital condyle variation with altered articulation at the craniovertebral junction [4,5,8] which in some cases may result in neck pain, reduced range of motion, or compensatory joint changes. In the present case, however, no macroscopic evidence of degenerative joint disease was observed, suggesting that the anatomical variation did not result in significant functional impairment or skeletal remodeling.

Together, these findings highlight the importance of distinguishing between non-pathological anatomical variation and biologically meaningful indicators of disease. The integration of osteological and molecular data provides a more nuanced understanding of individual health, demonstrating how inherited conditions and environmental stressors may interact to shape skeletal outcomes in past populations [1,13].

Within the archaeological context of the Sekköy assemblage, the analyzed individual should be interpreted as a single bioarchaeological case situated within a broader archaeological setting, rather than as representative of community-wide biological patterns [24].

The coexistence of porotic hyperostosis with a genetically confirmed heterozygous HbS mutation provides important biological context but does not establish hereditary hemoglobinopathy as a primary etiological factor [1,13]. Although porotic hyperostosis has traditionally been associated with anemia-related conditions, it is now widely recognized as a multifactorial skeletal indicator influenced by nutritional deficiency, infectious burden, environmental stress, and hereditary biological factors [10,11,12,19]. In the present case, the heterozygous HbAS genotype corresponds to sickle cell trait rather than homozygous sickle cell disease (HbSS), and would therefore not generally be expected to produce severe hematological manifestations under normal physiological conditions [17,19]. Consequently, the observed cranial lesions cannot be directly attributed to the HbS mutation alone.

Ancient DNA studies have demonstrated that hemoglobinopathies, including HbS and thalassemia-related variants, were present in archaeological populations from malaria-endemic regions of the Mediterranean and Near East [15,17,38,39]. These variants are widely interpreted as evolutionary adaptations to infectious disease pressure, particularly malaria, reflecting a balance between selective advantage and pathological consequence [15,16,17,38,39]. Previous studies have also shown that hereditary hemoglobinopathies may be associated with skeletal manifestations such as cranial porosity and marrow expansion under chronic hematopoietic stress [17,19]. However, given the heterozygous carrier status identified in the present individual, the findings are more appropriately interpreted within a cautious multifactorial paleopathological framework rather than as evidence of direct causation.

In contrast, the third occipital condyle identified in this individual is generally regarded as a rare non-pathological anatomical variant contributing to the spectrum of craniovertebral morphological diversity observed in past populations [3,9]. Clinical and anatomical studies have suggested that such variations may influence craniovertebral biomechanics and, in some cases, be associated with symptoms such as neck pain or altered articulation [4,6]. However, no clear macroscopic evidence of advanced degenerative joint disease, osteophyte formation, or articular surface erosion was observed in the present case, suggesting that any functional impact was likely limited [3,9]. Similar cranial base anomalies reported in Mediterranean and Near Eastern skeletal series further support their interpretation as developmental anatomical variants rather than pathological lesions [1,38].

Taken together, the combined occurrence of a rare craniovertebral anatomical variation, porotic hyperostosis, and a genetically confirmed HbS mutation represents a rare but informative bioarchaeological case. These findings highlight the importance of distinguishing non-pathological anatomical variation from biologically meaningful indicators of disease, while demonstrating the value of integrating osteological and molecular evidence to refine interpretations of health and adaptation in ancient populations [1,13,16].

Previous studies have shown that individuals affected by hemoglobinopathies frequently exhibit skeletal manifestations such as cranial porosity, marrow expansion, and dental alterations, all of which are linked to chronic hematopoietic stress [17,19]. The heterozygous state (HbAS) suggests a carrier condition rather than sickle cell disease, and therefore does not alone account for severe hematological stress.

The combined molecular and osteological findings should be interpreted cautiously within a multifactorial paleopathological framework. Although the detection of the heterozygous HbAS genotype provides relevant biological context, this carrier state does not support a direct causal attribution of the observed skeletal lesions to hereditary hemoglobinopathy alone [19]. Instead, the coexistence of porotic hyperostosis and HbAS should be understood in the broader context of interacting nutritional, infectious, environmental, and biological stressors that may have influenced skeletal remodeling in this individual [10,11,12,19]. This integrative interpretation is consistent with observations from other ancient populations and further highlights the value of incorporating molecular genetic evidence into paleopathological assessment [13,16,38,39].

The inferred Y-chromosomal haplogroup R1b (predicted based on Y-STR data) provides contextual information regarding the paternal lineage of the analyzed individual within the broader population dynamics of ancient Anatolia. Previous ancient DNA studies have documented the presence of R1b-related paternal lineages in the region from the Bronze Age onward, with continued representation into the Classical and Roman periods, contributing to the genetic diversity of Anatolian populations [27,40,41,42,43,44,45,46,47,48].

However, it is important to emphasize that haplogroup inference based on Y-STR data provides an approximate estimation rather than a definitive classification when compared to Y-SNP-based analyses. Accordingly, the haplogroup identified in this study should be interpreted with caution, and the results should be viewed as indicative of general population patterns rather than precise phylogenetic placement [20,23,37].

Within these limitations, the observed Y-STR profile is consistent with previously reported patterns of paternal lineage diversity in the region and likely reflects broader processes of population continuity and interaction rather than a specific or isolated demographic event. Haplogroup R1b and its derived subclades are widely distributed across Western Eurasia and have been documented in Anatolia from the Bronze Age onward at varying frequencies [29,30,31]. In this context, the inferred haplogroup in the present study aligns with established regional genetic patterns and supports interpretations of Anatolia as a long-standing zone of population interaction and genetic diversity.

Ancient DNA studies indicate that haplogroup R1b and its ancestral lineages have a broad temporal and geographical distribution across Western Eurasia [29,30,31,32,46,47]. Although their ultimate origins are often traced to Upper Paleolithic populations, their relevance in the present study lies in their documented presence in Anatolia from later prehistoric periods onward, reflecting long-term population movement and genetic interaction in the region [29,30,31].

**Figure 5:**
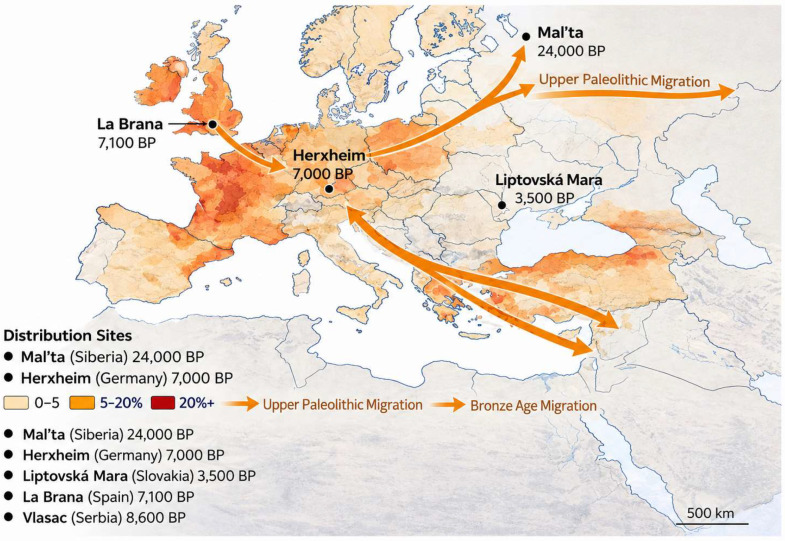
Simplified schematic representation of the geographical distribution of Y-chromosomal haplogroup R1b-related lineages across Western Eurasia, with emphasis on Anatolia. The figure is intended for illustrative purposes only and is based on previously published ancient DNA studies. It does not represent original analytical data or precise migration routes [29,30,31,32,33,34,35].

Ancient DNA studies have shown that R1b-related paternal lineages expanded across Europe during the Late Neolithic and Bronze Age, reflecting major demographic transformations associated with increased mobility and gene flow [29,33,35,36,48,49,50]. These lineages were also present in Anatolia from the Bronze Age onward, albeit at lower frequencies compared to dominant haplogroups such as J and G, indicating sustained gene flow and population interaction across the region [31,34]. In this context, the inferred Y-chromosomal haplogroup in the analyzed Roman-period individual from Sekköy is consistent with previously reported patterns of regional genetic diversity and supports the interpretation of Anatolia as a long-standing zone of population interaction rather than an isolated demographic unit. The broader geographical distribution of R1b-related lineages across Western Eurasia is summarized in Figure 5.

A limitation of this study is the absence of mitochondrial DNA (mtDNA) and genome-wide analyses, such as principal component analysis (PCA), which would provide a more comprehensive reconstruction of genetic ancestry. Due to the targeted nature of the sequencing strategy and preservation constraints, it was not possible to obtain sufficient data for these analyses. Future studies integrating genome-wide and maternal lineage data will be essential to refine interpretations of population affinity and biological relatedness. This study does not aim to provide population-level conclusions but rather presents a high-resolution case study illustrating the potential of integrative bioarchaeological approaches.

## 5. Conclusions

This study presents an integrated bioarchaeological and paleogenetic analysis of a Roman-period individual from the Sekköy site in southwestern Anatolia. The combined identification of a rare craniovertebral anatomical variation, skeletal indicators of physiological stress, and a genetically confirmed hemoglobin S (HbS) mutation provides a coherent framework for interpreting the interaction between morphology, disease, and genetic inheritance in antiquity.

The coexistence of porotic hyperostosis and a heterozygous HbS mutation (HbAS) supports a cautious multifactorial interpretation of skeletal stress, rather than a direct causal attribution to hereditary hemoglobinopathy alone. In parallel, the inferred Y-chromosomal haplogroup provides limited contextual information regarding paternal lineage affiliation within the broader archaeological—genetic framework of Anatolia.

Taken together, these findings highlight the value of integrating osteological and molecular data to reconstruct individual health profiles in archaeological contexts. This case demonstrates the methodological potential of interdisciplinary bioarchaeological approaches without supporting direct population-level inference.

## Data Availability

The nucleotide sequence generated in this study is available in GenBank under accession number PZ306518. Y-STR haplotype data and primer sequences are provided as Supplementary Materials.

## Supplementary Materials

The following supporting information can be downloaded at: https://www.mdpi.com/article/10.3390/life16060893/s1, Table S1: Primer sequences used for HBB amplification; Table S2: Y-STR haplotype profile of the analyzed individual; Table S3: Ancient DNA authentication and sequencing quality metrics.

## Abbreviations

The following abbreviations are used in this manuscript:

DNA	Deoxyribonucleic acid
aDNA	Ancient DNA
HBB	Hemoglobin beta
HbS	Hemoglobin S
STR	Short tandem repeat
PCR	Polymerase chain reaction
NGS	Next Generation Sequencing
UDG	Uracil–DNA glycosylase
UDG-half	Partial uracil–DNA glycosylase treatment
EDTA	Ethylenediaminetetraacetic acid
BWA	Burrows–Wheeler Aligner
PCA	Principal component analysis
mtDNA	Mitochondrial DNA
AMTL	Antemortem tooth loss
