# Supplementary material for: Integrating Osteology and Ancient DNA: Cranial Variation, Hemoglobin S, and Paternal Lineage in a Roman-Period Individual from Anatolia
Source: Life (Basel). 2026 May 26;16(6):893. doi: 10.3390/life16060893 (PMC13302398; doi:10.3390/life16060893)
Supplement: Supplementary file 1 [file life-16-00893-s001.zip › life-4284337-supplementary.pdf]

Supplementary Materials

Supplementary Table S1. Primer sequences used for amplification of the HBB gene region containing the HbS mutation (codon 6, rs334).

Primer Name	Direction	Sequence (5′–3′)	Target Region	Amplicon Size (bp)
HBB_F	Forward	ACTCCTGAGGAGAAGTCTGC	HBB exon 1 (codon 6 region)	120
HBB_R	Reverse	GAGTGGACAGATCCCCAAAG	HBB exon 1 (codon 6 region)	120

Primers were designed to amplify a short fragment of the HBB gene suitable for degraded ancient DNA. Amplicon length was kept below 150 bp to ensure efficient amplification of fragmented DNA.

Supplementary Table S2. Y-chromosomal STR haplotype of the analyzed individual.

Locus	Allele
DYS456	12
DYS389I	13
DYS390	24
DYS389II	30
DYS458	17
DYS19	15
DYS385a	11
DYS385b	14
DYS393	13
DYS391	11
DYS439	12
DYS635	23
DYS392	13
GATAH4	12
DYS437	15
DYS438	12
DYS448	20

Only alleles that were reproducible across independent amplifications were included in the final haplotype to minimize stochastic effects associated with degraded ancient DNA.

Supplementary Information

The nucleotide sequence obtained in this study has been deposited in GenBank under accession number PZ306518.

The Y-STR haplotype profile is provided in full to ensure transparency and reproducibility of the genetic analyses performed in this study.

Supplementary Table S3. Ancient DNA sequencing quality, authentication, and rs334 locus-specific metrics for the analyzed individual

**Metric**	**Value**
Total raw reads	142,061,360
Successfully mapped reads	135,400,927 (98.42%)
Uniquely mapped reads	130,272,327 (96.21%)
Average target coverage	136.6×
Q20 score	97.93%
Q30 score	93.92%
Effective data yield after preprocessing	82.44%
Ancient DNA authentication	Characteristic terminal nucleotide misincorporation patterns consistent with authentic ancient DNA, as confirmed by mapDamage analysis
Endogenous human DNA content	98.42%
HbS locus (rs334) read depth	38×
rs334 mutant allele read count	18
rs334 wild-type allele read count	20
HbS locus (rs334) interpretation	Variant supported by independent sequencing reads and confirmed by Sanger sequencing
Genotype interpretation	Heterozygous HbAS (one mutant allele, one wild-type allele)
